# Incomplete renal tubular acidosis as a predisposing factor for calcium phosphate stones in neuropathic bladder: a case report

**DOI:** 10.1186/1757-1626-1-318

**Published:** 2008-11-17

**Authors:** Subramanian Vaidyanathan, Bakul M Soni, Ian D Watson, Gurpreet Singh, Peter L Hughes, Paul Mansour

**Affiliations:** 1 Regional Spinal Injuries Centre, District General Hospital, Southport, PR8 6PN, UK; 2 Department of Clinical Biochemistry, District General Hospital, Southport, PR8 6PN, UK; 3 Department of Urology, District General Hospital, Southport, PR8 6PN, UK; 4 Department of Radiology, District General Hospital, Southport, PR8 6PN, UK; 5 Department of Cellular Pathology, District General Hospital, Southport, PR8 6PN, UK

## Abstract

We present a male tetraplegic patient, who developed stones in neuropathic bladder six times within a span of three years. Unusual features of this case are: (1) This patient started developing stones in urinary bladder thirteen years after sustaining spinal cord injury. (2) He was performing intermittent catheterisation and did not have an indwelling catheter. (3) The presenting symptom of vesical lithiasis was abdominal spasms and not urine infection. (4) The major component of the stones was calcium phosphate; magnesium ammonium phosphate was completely absent in the calculus on four occasions. (5) Proteus species were not grown from urine at any time. (6) This patient failed to acidify urine below a pH of 5.3 after taking simultaneously furosemide (40 mg) and fludrocortrisone (1 mg), which suggested incomplete renal tubular acidosis type 1.

We learn from this case that biochemical analysis of stones removed from urinary bladder may be useful. If the major component of vesical calculus is calcium phosphate, complete or incomplete renal tubular acidosis type 1 should be excluded, as it may be possible to reduce the risk of recurrence of calcium phosphate stones by oral potassium citrate therapy or, vegetable and fruit rich diet.

## Background

A study of bladder calculi in 500 persons treated at the University of Alabama in Birmingham Spinal Cord Injury Care System between 1973 and 1981 showed that bladder calculi were most likely to develop within one year of spinal cord injury. Patients developing bladder calculi prior to first definitive discharge were most likely to have neurologically complete lesions and Klebsiella infections at admission. Patients developing bladder stones within two years of hospital discharge were most likely to have indwelling urethral catheters and either Proteus or multiple-organism infections at discharge. [1] Thus indwelling urinary catheter and urine infections are major risk factors for vesical calculi in spinal cord injury patients. Patients, who develop vesical calculi, often present with catheter blockages or urine infections. These stones usually contain magnesium ammonium phosphate. When a spinal cord injury patient develops recurrent urine infections and vesical calculi, squamous metaplasia may be seen in the neuropathic bladder. Spinal cord injury patients, who develop recurrent nephrolithiasis, are advised to undergo biochemical evaluation, but patients with vesical calculi do not usually receive such advice, as metabolic abnormalities are uncommon in those who form stones only in the urinary bladder. Similarly, dietary modification or oral citrate therapy is recommended to patients with calcium phosphate kidney stones and not for those with bladder stones.

We report an adult male patient with tetraplegia, who developed recurrent bladder calculi. This case had several unusual features. The presenting symptom of vesical calculus was abdominal spasm. This patient was performing intermittent catheterisations and did not have long-term catheter drainage. He started developing bladder stones thirteen years after sustaining spinal cord injury. The major component of vesical calculi was calcium phosphate. Renal acidification test revealed incomplete renal tubular acidosis type 1.

## Case presentation

A Caucasian male, born in 1943, sustained fracture dislocation of seventh cervical and first thoracic vertebrae with tetraplegia in 1992, when he lost control of motorbike. Internal fixation and fusion of cervico-dorsal junction was performed. He was managing his bladder by a penile sheath. In 1997, this person was taught the technique of self-catheterisation. He started performing intermittent catheterisations.

Intravenous urography was performed as a routine test on 06 August 1997, and it showed marked prostatic calcification. Kidneys, ureters and bladder were normal. Intravenous urography was performed during a routine follow-up on 30 June 1999; and no radio opaque calculi were seen. Both kidneys excreted the contrast; normal kidney, pelvicalyceal systems, ureters and bladder. As the patient developed frequent urine infections, intravenous urography was performed on 31 January 2000. No radio opaque renal or vesical calculi were seen; prostatic calcification was present. Both kidneys excreted contrast; normal kidneys, pelvicalyceal system, ureters and bladder. There was no significant residual urine.

In September 2004, this person developed spasms of abdomen, cold sweats and headache. He was moving his bowels every day in the evening and was using Carbalax suppositories. Intravenous urography was performed on 05 November 2004. Kidneys and ureters were normal. Multiple diverticula arising from the bladder were noted.

**Figure 1:**
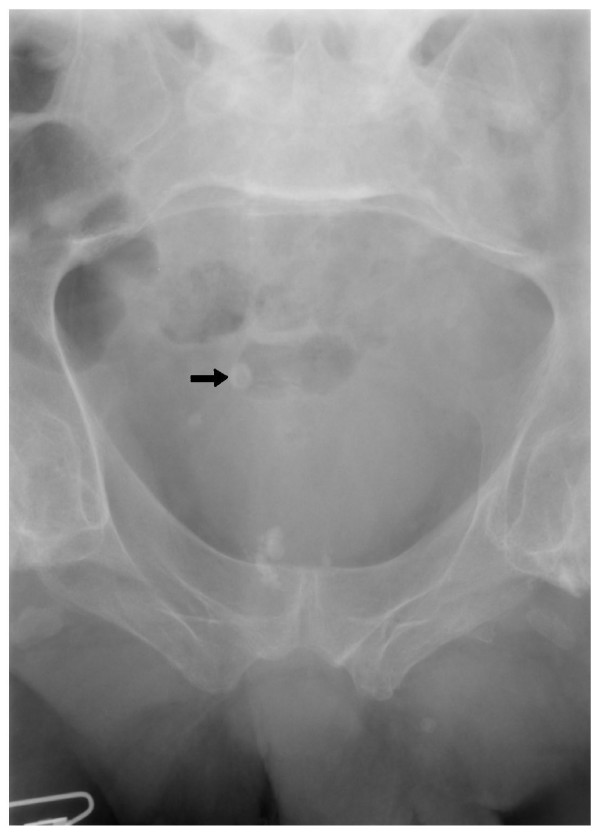
**X-ray of urinary bladder taken on 07 March 2005 shows prostatic calcification.** There is a radio opaque shadow in the region of urinary bladder, which is probably a vesical calculus (arrow).

**Figure 2:**
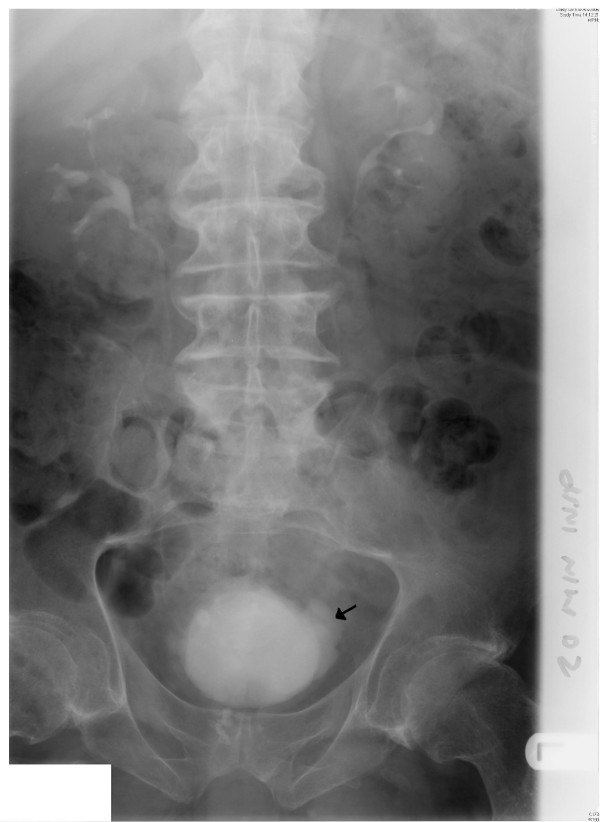
Intravenous urography (07 March 2005): twenty minutes film shows undilated pelvicalyceal systems on both sides; multiple, small diverticulae arise from the urinary bladder (arrow).

This patient attended spinal unit on 24 January 2005 with spasm of abdomen. Chest X-ray, which was taken on 04 March 2005, showed clear lungs. X-ray of abdomen revealed moderate faceal loading of the colon. Ultrasound scan of abdomen showed multiple gallstones; common bile duct was not dilated. Ultrasound examination of urinary tract revealed a simple cyst in the upper pole of right kidney; no renal calculi; no renal scarring or hydronephrosis. There was a calculus in the urinary bladder. Intravenous urography revealed prostatic calcification and a vesical calculus. (Figure 1) Pelvicalyceal systems and ureters were normal. Diverticula were seen in the bladder. (Figure 2) On 18 November 2005, this person underwent external urethral meatotomy, cystoscopy and elctrohydrualic lithotripsy of vesical calculi. (Table 1) Following removal of stones from urinary bladder, spasm of abdomen disappeared and this person could sleep during night.

**Table 1:** List of dates when cystoscopy and electrohydraulic lithotripsy of vesical calculi were performed

**Date**	Procedure
18 November 2005	Tight external urethral meatus: meatotomy was performed with Otis urethrotome. Electrohydrualic lithotripsy of vesical calculi was carried out. Fragments of stones were removed completely.
08 December 2006	Electrohydrualic lithotripsy of vesical calculi was carried out. Fragments of stones were removed completely.
18 May 2007	Electrohydrualic lithotripsy of vesical calculi was carried out. Fragments of stones were removed completely.
02 November 2007	Electrohydrualic lithotripsy of vesical calculi was carried out. Fragments of stones were removed completely.
18 April 2008	Electrohydrualic lithotripsy of vesical calculi was carried out. Fragments of stones were removed completely.
27 June 2008	Electrohydrualic lithotripsy of vesical calculi was carried out. Fragments of stones were removed completely.

**Figure 3:**
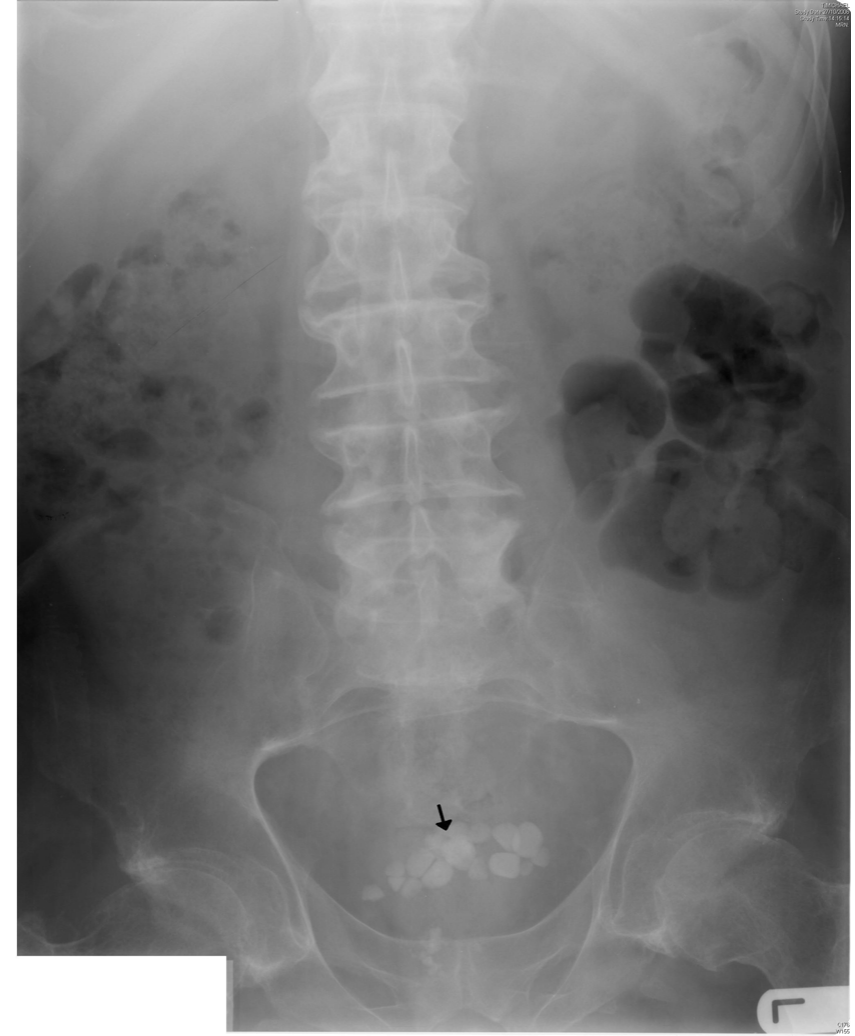
X-ray of abdomen (27 October 2006) reveals several calcified stones in urinary bladder (arrow).

**Figure 4:**
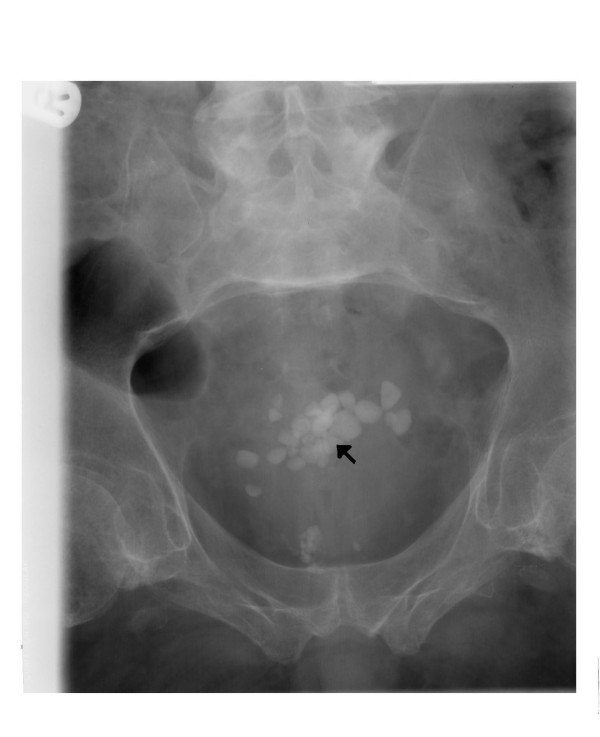
X-ray of urinary bladder taken on 14 May 2007 shows several vesical calculi (arrow).

In August 2006, he developed severe spasms in the stomach. X-ray of abdomen revealed several calculi in urinary bladder. (Figure 3) Cystoscopy and electrohydraulic lithotripsy were carried out on 08 December 2006. (Table 1) In May 2007, he developed spasms of abdomen once again. Not surprisingly, X-ray of abdomen showed several stones in urinary bladder. (Figure 4) Electrohydraulic lithotripsy was carried out on 18 May 2007. The stones were very hard to break. This observation, which was made during lithotripsy, corroborated with the biochemical analysis of the stone. (Table 2) The stone was found to contain 100% calcium phosphate.

**Table 2:** Results of biochemical analysis of stones, which were removed from urinary bladder: composition of stone is shown in percentage

**Date when a sample of calculus removed from urinary bladder was received by Biochemistry Laboratory for analysis**	**Weight of the specimen (milligrams)**	**Calcium Oxalate**	**Calcium Phosphate**	**Magnesium ammonium phosphate**
21 November 2005	64	Absent	64%	36%
08 December 2006	7184	6%	94%	Absent
18 May 2007	5737	Absent	100%	Absent
02 November 2007	43	9%	90%	Absent
21 April 2008	1149	Absent	88%	11%
27 June 2008	87	10%	90%	Absent

**Figure 5:**
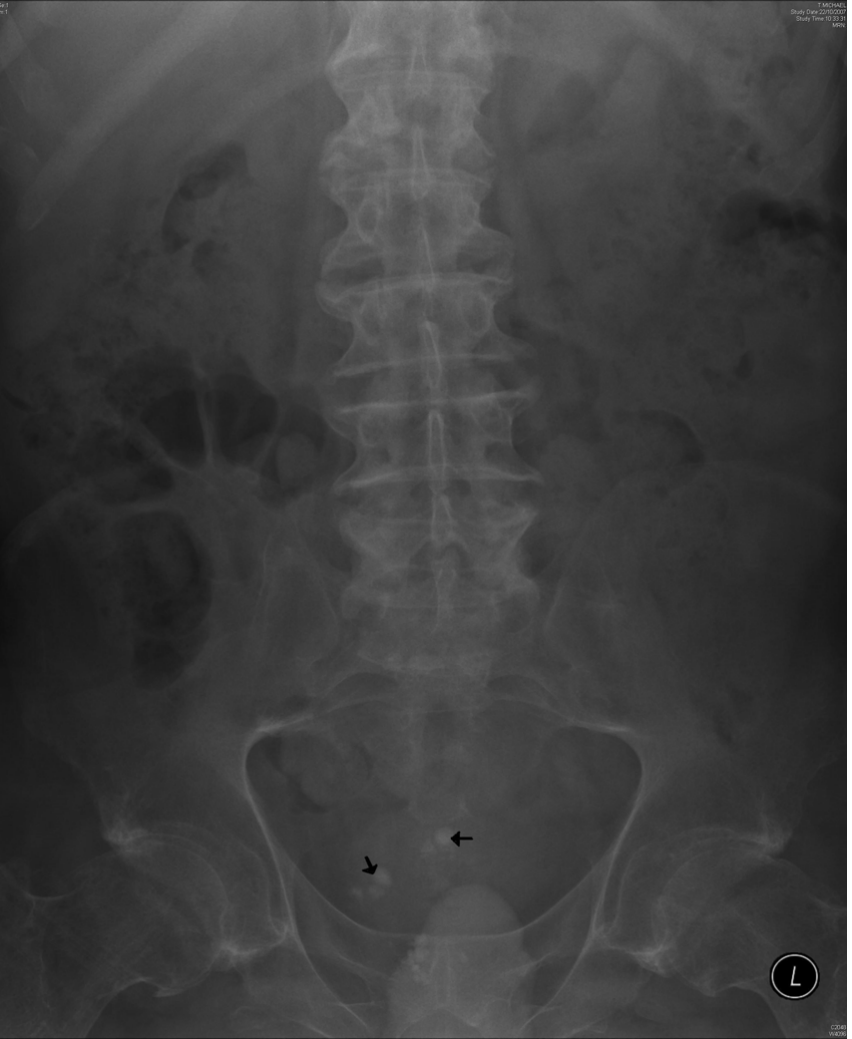
X-ray of abdomen taken on 22 October 2007 shows the presence of small stones in urinary bladder (arrow).

This patient again developed spasms of abdomen in September 2007. X-ray of abdomen showed vesical calculi. (Figure 5) Electrohydraulic lithotripsy was performed on 02 November 2007. All fragments of stones were removed from the bladder. Following the procedure, a pressure mark over sacrum was noticed. This was non-blanchable erythema of intact skin, which represented Grade 1 pressure ulcer. The patient was advised to stay in bed and lie on his sides until the pressure mark healed completely.

**Figure 6:**
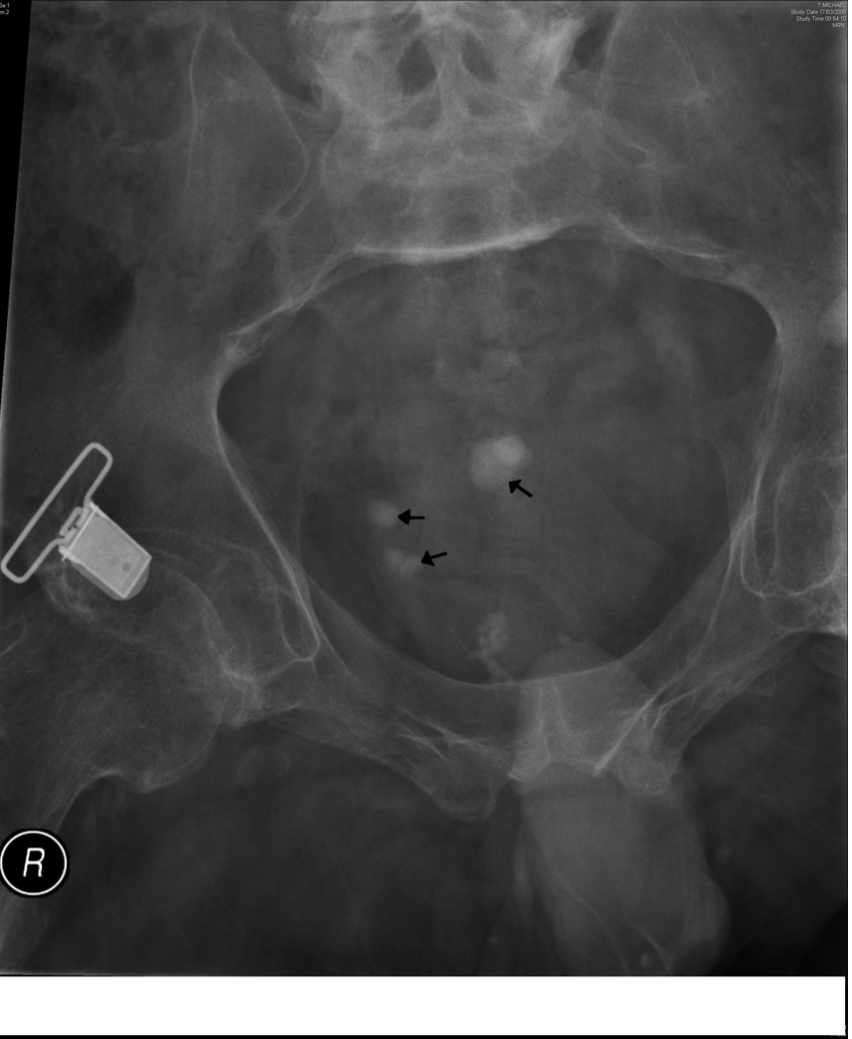
X-ray of urinary bladder (17 March 2008) shows four or five small stones (arrow).

**Figure 7:**
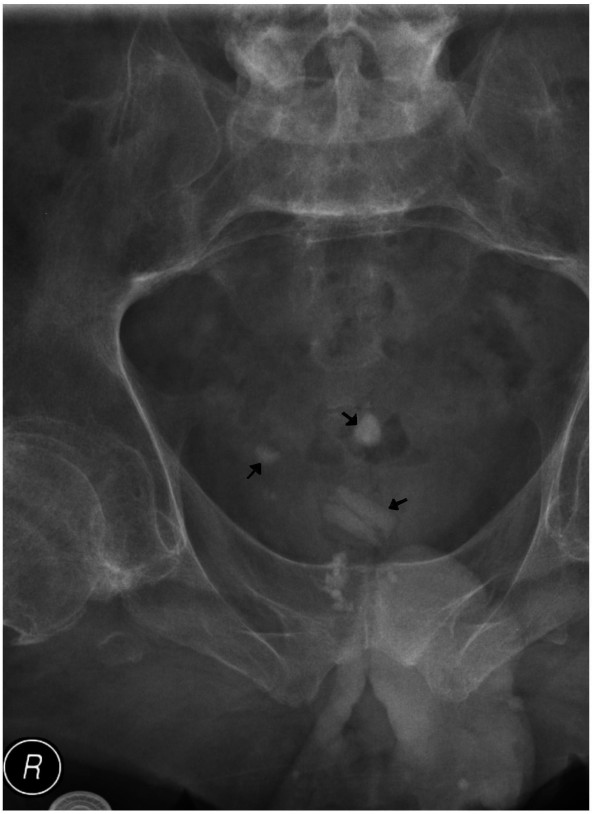
X-ray of urinary bladder taken on 09 June 2008 reveals one large and two small calculi (arrow).

He attended spinal unit on 26 March 2008 with abdominal spasms. Radiograph of abdomen revealed several vesical calculi. (Figure 6) Electrohydraulic lithotripsy was carried out on 18 April 2008. It was ensured that no piece of stone was left behind in the bladder or in vesical diverticula. He did very well after lithotripsy. But this patient came back on 09 June 2008 with abdominal spasms. X-ray of abdomen showed stones in urinary bladder. (Figure 7) Electrohydraulic lithotripsy was performed on 27 June 2008. This patient did well after the sixth operation for removal of recurrent vesical calculi.

**Figure 8:**
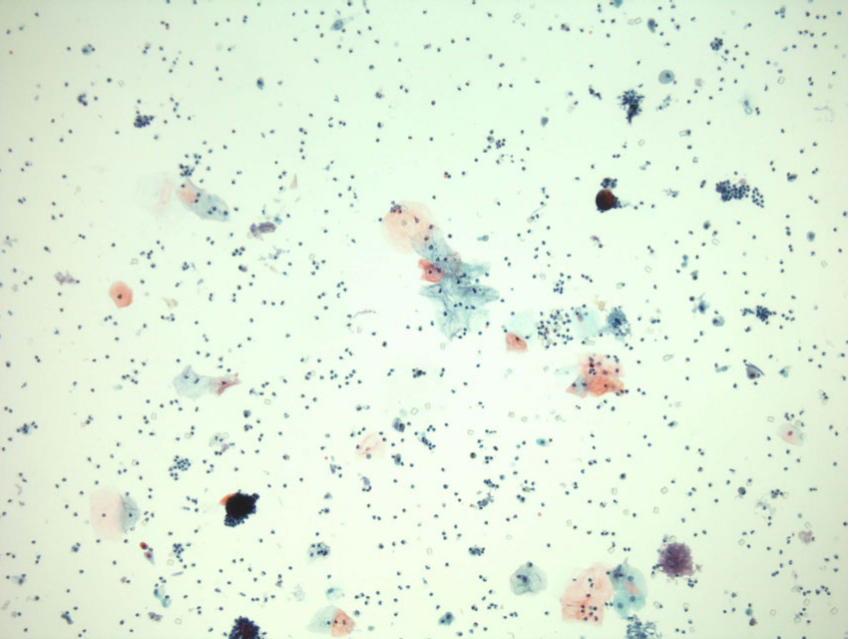
**Urine cytology photomicrograph shows benign epithelial cells with a background of neutrophil ploymorphs.** (Cytospin preparation, Papanicolau stain).

**Figure 9:**
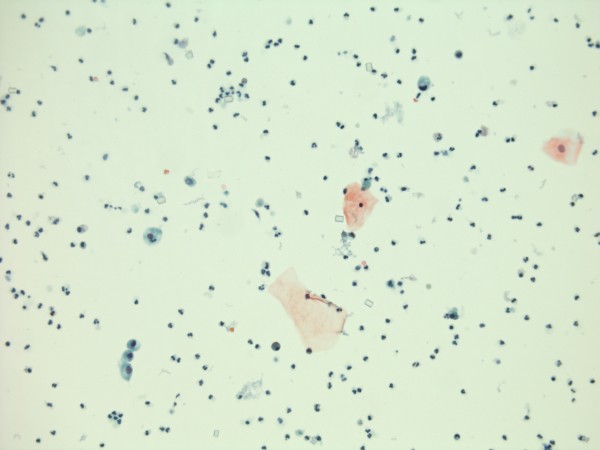
**Urine cytology: higher power view shows benign urothelial cells (left middle and left bottom) and squamous cells (middle and middle right).** No keratinising squamous cells are present. (Cytospin preparation, Papanicolau stain)

Since bladder stone disease is a risk factor for vesical malignancy in patients with spinal cord injury, a sample of urine was sent for cytology. Cytology showed benign epithelial cells; no suspicious or malignant cells were seen. No keratinising squamous cells were identified. (Figures 8 and 9)

This patient performed intermittent catheterisations about six times a day; he preferred ready to use, sterile, pre-lubricated SpeediCath catheters size 12 French (Coloplast Ltd, Peterborough PE2 6FX, United Kingdom) for self-catheterisations because the catheter package already contained water for lubrication and it was user-friendly. With regular intermittent catheterisations, this patient remained continent and was able to discard penile sheath and leg bag. Asymptomatic bacteriuria was observed on several occasions and organisms isolated from urine are listed in Table 3

**Table 3:** Results of urine microbiology

**Date**	**Bacteria grown from culture of urine**	**Clinical symptoms**
27 September 1992	Enterococcus faecalis	Persistent fever despite gentamicin
07 June 1999	*Klebsiella aerogenes*	Cold flushes down the back
22 September 1999	Klebsiella aerogenes	Smelly urine
01 June 2000	Pseudomonas aeruginosa	Feeling unwell
22 March 2005	Escherichia coli	Abdominal spasms
16 August 2005	Escherichia coli	Abdominal spasms
07 November 2005	*Klebsiella aerogenes *and *Escherichia coli*	Pre-operative assessment
14 November 2005	*Klebsiella aerogenes *and *Escherichia coli*	Pre-operative assessment
19 January 2006	Enterococcus faecalis	Spasms in stomach
04 August 2006	*Pseudomonas aeruginosa *and *Enterococcus faecalis*	Increased spasms
03 November 2006	*Enterococcus *species and *Pseudomonas *species	Pre-operative assessment
14 May 2007	*Escherichia coli *and *Enterococcus faecalis*	Pre-operative assessment
29 October 2007	*Escherichia coli *and *Enterococcus faecalis*	Pre-operative assessment
19 March 2008	*Escherichia coli *and *Enterococcus faecalis*	Pre-operative assessment
07 April 2008	Escherichia coli	Pre-operative assessment
23 June 2008	Escherichia coli	Pre-operative assessment

Since this patient developed repeatedly stones in urinary bladder, we carried out biochemical tests of blood and urine to look for underlying metabolic abnormality, if any. When biochemical tests were carried out, this patient had started taking orange juice in large quantities in the hope of preventing further formation of urinary stones. Results of biochemical tests are given below. Reference range is mentioned within parenthesis.

## 24-hours urine

14 November 2005 Urine volume: 1102 mL

14 November 2005 Calcium: 5.08 mmol/day (1.50 – 2.50)

14 November 2005 Urate: 2.97 mmol/day (1.19 – 2.98)

14 November 2005 Citrate: 2.18 mmol/24 hours (1.68 – 6.45)

14 November 2005 Oxalate: 187 umol/day (189 – 477)

16 November 2005 Urine volume: 1120 mL

16 November 2005 Creatinine clearance: 71 mL/minute

15 May 2007 Urine volume: 1360 mL

15 May 2007 Calcium:5.47 mmol/day (2.50 – 7.50)

15 May 2007 Phosphate: 20.3 mmol/day (12.9 – 42.0)

15 May 2007 Urate: 1.43 mmol/day (1.19 – 2.58)

01 April 2008 Urine volume: 1050 mL

01 April 2008 Oxalate: 312 umol/day (189–477)

01 April 2008 Urate: 0.87 mmol/day (1.19–2.98)

01 April 2008: Calcium: 3.85 mmol/day (2.50–7.50)

09 April 2008 Urine volume: 1244 mL

09 April 2008 Citrate: 2.24 mmol/24 hours (1.68–6.45)

03 June 2008 Urine volume: 1480 mL

03 June 2008 Calcium: 4.53 mmol/day (2.50–7.50)

03 June 2008 Oxalate: 388 umol/day (189–477)

03 June 2008 Urate: 2.96 umol/day (1.19–2.98)

18 June 2008 Urine volume: 1150 mL

18 June 2008 Calcium: 4.14 mmol/day (2.50–7.50)

18 June 2008 Urate: 0.97 umol/day (1.19–2.98)

08 July 2008 Urine volume: 1860 mL

08 July 2008 Oxalate: 370 umol/day (189–477)

## Blood biochemistry

Calcium: 2.23 mmol/L (2.20–2.60)

Phosphate: 1.01 mmol/L (0.80–1.50)

Alkaline phosphatase: 106 u/L (40–120)

Sodium: 139 mmol/L (133–146)

Potassium: 4.3 mmol/L (3.5–5.2)

Chloride: 105 mmol/L (95–110)

Bicarbonate: 34 mmol/L (22–30)

Magnesium: 0.96 mmol/L (0.70–1.00)

Urea: 6.5 mmol/L (3.0–9.0)

Creatinine: 58 umol/L (0–150)

Urate: 0.20 mmol/L (0.00–0.43)

Random glucose: 6.9 mmol/L (3.6–7.8)

TSH: 2.000 iu/L (0.300–5.00)

Free T 4: 12.4 pmol/L (9.0–24.0)

C-terminal telopeptide:0.32 ng/ml (0.10–0.50)

## Urine biochemistry

Random urine bicarbonate: 2.3 mmol/L

Random urine bicarbonate (12 August 2008): 1.7 mmol/L

Urine pH: 6.1

In summary, this patient had citrate level in urine at the bottom of the reference range. Urine pH was 6.1, which would be conducive to calcium phosphate stone formation. We carried out urinary acidification test to look for distal renal tubular acidosis after taking written informed consent from the patient.

Urinary acidification was assessed by simultaneous administration of furosemide (40 mg) and fludrocortisone (1 mg). A baseline urine sample was taken followed by oral administration of furosemide (40 mg) and fludrocortisone (1 mg). Fluid intake was *ad libitum*. Urine was collected hourly for the next six hours. The results of urine acidification test are given in Table 4. The minimum pH value of urine was 5.56, which was noted four hours after ingestion of furosemide and fludrocortisone. Failure to acidify urine to a pH of less than 5.3 is consistent with incomplete distal renal tubular acidosis.

**Table 4:** Results of urine acidification test: furosemide 40 mg and fludrocortisone 1 mg were administered by mouth at 1000 hours

**Time (hours)**	**Urine pH**	**Urine Sodium (mmol/L)**	**Urine Potassium (mmol/L)**	**Urine Urea (mmol/L)**
0945	6.3	31	20	85
1100	6.0	106	36	85
1200	6.3	74	22	23
1300	5.7	47	21	21
1400	5.56	49	35	44
1500	7.4	23	135	150
1600	7.5	16	161	212

Therapeutic options were: (1) to prescribe potassium citrate or sodium citrate; (2) to advise the patient to take vegetables and fruits daily. Both potassium citrate and sodium citrate were available on prescription and therefore, the patient did not have to pay for these medicines. But the patient would have to buy fruits, vegetables or fruit juices. We were concerned about the possible side effect of sodium citrate therapy, as supplementation of sodium can potentially affect blood pressure especially when taken for a prolonged period. The patient decided to take more citrus fruits in his diet every day. He was given information sheet on citrate content of different fruits and drinks. [2]

## Discussion

A review of the medical records of all veterans with spinal cord injury, who received care in Michael E DeBakey Veterans Affairs Medical center, Houston, Texas, USA from 1 January 2003 to 2 January 2006 [3] revealed that 71 subjects were diagnosed with *Proteus *in their urine at some point during the study period, and 19 of them (27%) were also diagnosed with stones (*P *= 0.045). Other organisms associated with the presence of stones included *Enterococcus, Klebsiella, Pseudomonas, Morganella*, and *Providencia *(*P *< 0.05). In this patient, Proteus was not grown in urine at any time, but this patient had asymptomatic bacteriuria with *Escherichia coli, Enterococcus faecalis, Klebsiella aerogenes*, and *Pseudomonas aeruginosa*.

Calcium phosphate calculi in urinary bladder are rare, as magnesium ammonium phosphate, which is associated with urine infection, is the common vesical calculus in patients, who have indwelling catheters. Calcium phosphate stones in kidneys are seen in patients with renal tubular acidosis type 1. Typically patients with complete renal tubular acidosis type 1 have metabolic acidosis, alkaline urine, decreased urinary citrate, and hypercalcuria. Persons with incomplete renal tubular acidosis type 1 may not markedly manifest any of these abnormalities. Investigation of this patient revealed little that was abnormal, however he was found to have a basal urine pH of 6.1, which would be conducive to calcium phosphate calculi formation. Therefore, we performed urinary acidification test by simultaneous administration of furosemide (40 mg) and fludrocortisone (1 mg). The short ammonium chloride test, which involves the oral ingestion of a quantity of ammonium chloride and serial measurements of urine pH, is adopted internationally as the 'gold standard' diagnostic test for distal renal tubular acidosis. However, although the ammonium chloride test lasts eight hours and does not require blood testing, it can be unpleasant for some patients, because gastric irritation, nausea, and vomiting are common. Walsh and associates [4] showed that simultaneous administration of furosemide (to increase distal tubular sodium delivery) and fludrocortisone (to enhance principal cell sodium re-absorption and alpha intercalated cell hydrogen ion secretion) provided a sufficient and consistent stimulus to unmask an acidification defect in distal renal tubular acidosis, without the need for ammonium chloride. Simultaneous administration of furosemide and fludrocortisone provided an easy, effective, and well-tolerated alternative to the standard ammonium chloride urinary acidification test for the diagnosis of distal renal tubular acidosis. Following administration of furosemide (40 mg) and fludrocortisone (1 mg), the urine pH decreased to less than 5.3 in all healthy volunteers whereas all patients with previously diagnosed distal renal tubular aciodosis failed to acidify their urine to pH less than 5.3. Ammonium chloride test and furosemide/fludrocortisone test assess urinary acidification in different ways: the former by providing an acid load for the distal nephron to excrete and the latter by producing direct and indirect stimulation of distal nephron hydrogen ion secretion.

When calcium phosphate calculi, which are rare, are found in urinary bladder, it is advisable to exclude complete or incomplete renal tubular acidosis type 1. In case a patient has renal tubular acidosis, it is possible to reduce the risk of recurrence of calcium phosphate stones by increasing citrate intake or taking vegetable and fruit rich diet. [5]

Spinal cord injury patients, who have indwelling catheters and develop vesical calculi, present with catheter blockages and urine infections. This patient was performing intermittent catheterisations and developed calcium phosphate stones in urinary bladder. The initial presenting symptom of vesical calculi in this patient was abdominal spasms. Doctors caring for spinal cord injury patients should be aware of the unusual symptoms, which are seen in spinal cord injury patients with various diseases. Hyperhidrosis may be the presenting symptom of renal calculi in a patient with spinal cord injury. [6]

This case raises an important question to which we do not have an answer.

➢ Why did this patient with incomplete renal tubular acidosis develop calcium phosphate stones in urinary bladder and not in the kidneys?

We shall submit a follow-up of this patient after 18 months to inform the readers whether the diet rich in vegetables and fruits has been useful in preventing recurrence of calcium phosphate stones in urinary bladder.

## Conclusion

We learn from this case that abdominal spasms may be the presenting feature of calcium phosphate stones in urinary bladder of spinal cord injury patients, as these stones, unlike magnesium ammonium phosphate stones, are not associated with recurrent urine infections. It is advisable to send specimens of stones removed from urinary bladder for biochemical analysis. If the major component of vesical calculus is found to be calcium phosphate, complete or incomplete renal tubular acidosis type 1 should be excluded, as it may be possible to reduce the risk of recurrence of calcium phosphate stones by oral potassium citrate therapy or vegetable and fruit rich diet.

## Consent

Written informed consent was obtained from the patient for publication of this case report and accompanying images. A copy of the written consent is available for review by the Editor-in-Chief of this journal.

## Competing interests

The authors declare that they have no competing interests.

## Authors' contributions

SV developed the concept and wrote the draft. IW supervised biochemistry investigations. All authors contributed to patient care.
